# Moisture effect on the diffusion of Cu ions in Cu/Ta_2_O_5_/Pt and Cu/SiO_2_/Pt resistance switches: a first-principles study

**DOI:** 10.1080/14686996.2019.1616222

**Published:** 2019-06-03

**Authors:** Bo Xiao, Satoshi Watanabe

**Affiliations:** a Department of Materials Engineering, The University of Tokyo, Tokyo, Japan; b Laboratory of Theoretical and Computational Chemistry, School of Chemistry and Chemical Engineering, Yantai University, Yantai, China

**Keywords:** Resistance switches, Cu ion diffusion, moisture effect, first-principles simulation, 40 Optical, magnetic and electronic device materials, 401 1st principle calculations

## Abstract

Cu/Ta_2_O_5_/Pt and Cu/SiO_2_/Pt are two of the most promising resistance switches. From experimental observations, it is speculated that the presence of H_2_O in the amorphous Ta_2_O_5_ and SiO_2_ (a-Ta_2_O_5_ and a-SiO_2_) facilitates the rate-limiting step during the switching process. This rate-limiting step is essentially the diffusion of Cu ions along the nanopores of the amorphous. To better understand this behavior and obtain a detailed examination of the atomic structures, a first-principles simulation was conducted. In addition, we investigate the diffusion behaviors of Cu ions in bare a-Ta_2_O_5_ nanopore and in the one covered with H_2_O–together with those in a-SiO_2_ nanopore. Our work reveals that Ta and Si atoms on the sidewalls of bare a-Ta_2_O_5_ and a-SiO_2_ nanopores are in the unsaturated (TaO_5_) and saturated (SiO_4_) forms, respectively. Consequently, H_2_O molecules are adsorbed on the nanopore sidewall strongly in the case of a-Ta_2_O_5,_ and weakly in a-SiO_2_, by forming O-Ta and H∙∙∙O bonds, respectively. This can explain the experimental observation that the desorption of H_2_O occurs only at high temperatures for a-Ta_2_O_5_ films, while it is observed for a-SiO_2_ even when the temperature is low. The calculated diffusion barrier of Cu ions in a-Ta_2_O_5_ nanopores covered with H_2_O is about 0.43 eV, which is much lower than that without H_2_O (~1.40 eV). In view of the similar chemical environments of O and the adsorbed Cu ions in a-SiO_2_ and a-Ta_2_O_5_ nanopores, it is expected that the diffusion of Cu ions in a-SiO_2_ nanopore without H_2_O is much more difficult than with H_2_O. This could be attributed to the strong and weak adsorption of Cu ions on the sidewall in the absence and presence of H_2_O, respectively, for both, a-Ta_2_O_5_ and a-SiO_2_. Our investigation provides a full atomic picture to understand the moisture effect on the diffusion of Cu ions in Cu/a-Ta_2_O_5_/Pt and Cu/a-SiO_2_/Pt resistance switches.

## Graphical Abstract

**Figure:**
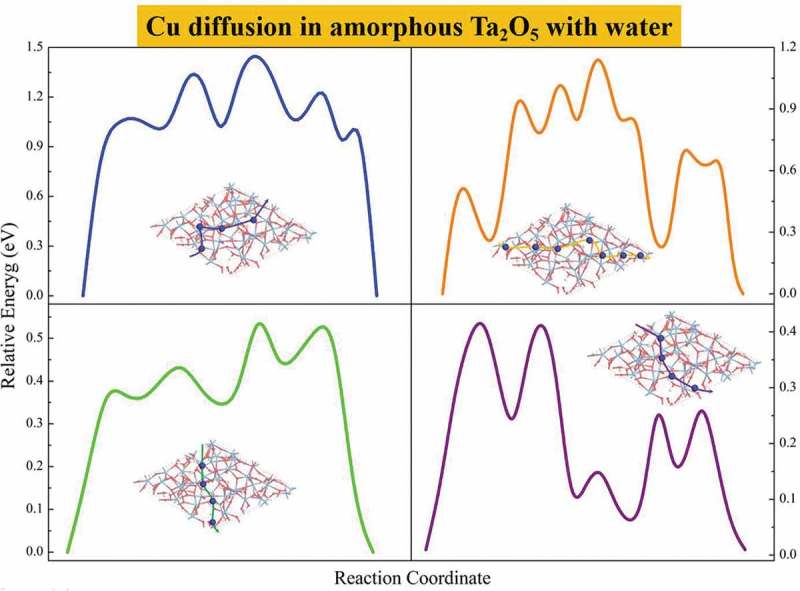

## 1. Introduction

Cation-based resistance switches or electrochemical metallization memories (ECM) have attracted significant attention due to their high scalability, low power consumption and potential application in memory cells [1,2]. In general, such devices consist of an insulator (such as, HfO_x_ [3], SiO_x_ [4], TaO_x_ [5], etc.) layer sandwiched between an inert electrode (Pt or Au) and an oxidizable electrode (Cu or Ag). Their switching processes (from high resistance state to low resistance state) could be realized by changing the polarity of the applied voltage between the electrodes.

Among the ECM devices, amorphous Ta_2_O_5_ and SiO_2_ (a-Ta_2_O_5_ and a-SiO_2_)-based devices (i.e. Cu/a-Ta_2_O_5_/Pt and Cu/a-SiO_2_/Pt) have been extensively studied considering their high performances [5,6]. Composition analyses of a-Ta_2_O_5_ and a-SiO_2_ films before and after applying a voltage reveal that the switching phenomenon is mainly due to the formation/rupture of Cu filaments [7,8]. Recently, the moisture effects on the performance of a-Ta_2_O_5_- and a-SiO_2_-based ECM devices have been studied experimentally [6,9]. The following are the key outcomes: (i) none of the devices could be formed without the presence of H_2_O, (ii) the forming voltage drops dramatically with increase in the content of H_2_O in a-Ta_2_O_5_ and a-SiO_2_ films, (iii) the operation voltage of a-Ta_2_O_5_ (a-SiO_2_)-based ECM device rarely (strongly) depends on the ambient H_2_O pressure.

During the switching processes of Cu/a-Ta_2_O_5_/Pt and Cu/a-SiO_2_/Pt ECM devices, three rate-limiting steps have been proposed in the literature. These are (i) ionization of Cu electrode at the Cu/a-Ta_2_O_5_ and Cu/a-SiO_2_ interfaces, (ii) diffusion of Cu ions in the a-Ta_2_O_5_ and a-SiO_2_, and (iii) nucleation of Cu ions at the Pt electrode. These key factors determine the performance of ECM devices (such as, the forming and operation voltages, endurance and switching rates). Accordingly, the ‘moisture effect’ on the performance of ECM devices can be attributed to the influence of the above-mentioned rate-liming steps due to the presence of H_2_O [6,9–11]. In particular, moisture has been found to be important in the processes of diffusion and resistive switching (e.g. the counter electrode reaction) [10,11]. Studies on moisture effect in such processes remain to be done. In the present work, we pay due attention on the ‘moisture effect’ on the diffusion of Cu ions in ECM devices. The following hypothesis is proposed on the basis of experimental observations: the nanoporous structures of the deposited a-Ta_2_O_5_ and a-SiO_2_ films have strong and weak moisture adsorption characteristics during ambient exposure [12,13], respectively. The Cu diffusion along the sidewalls of a-Ta_2_O_5_ and a-SiO_2_ nanopores becomes smoother when the nanopores are covered with H_2_O. The strong dependence of the operation voltage on the ambient H_2_O pressure in the case of a-SiO_2_ can be understood from weaker adsorption of H_2_O on the sidewall of a-SiO_2_. However, several issues need to be addressed: (i) what is the atomic structures of a-Ta_2_O_5_ and a-SiO_2_ nanopores, (ii) why is the adsorption behavior of H_2_O in a-Ta_2_O_5_ and a-SiO_2_ nanopores significantly different, (iii) how the Cu ions diffuse along the a-Ta_2_O_5_ and a-SiO_2_ nanopores with and without the presence of H_2_O. Accordingly, the atomic-scale simulations on the structures of a-Ta_2_O_5_ and a-SiO_2_ nanopores, and the Cu diffusion behaviors in these nanopores would be very helpful not only to understand the switching process of ECM devices, but also to design the devices for high performance.

In this work, by using the first-principles simulation, we examine the atomic structures and Cu ions diffusion behaviors in a-Ta_2_O_5_ and a-SiO_2_ nanopores with and without the presence of H_2_O. Our work shows that the different adsorption behaviors of H_2_O in a-Ta_2_O_5_ and a-SiO_2_ nanopores can be ascribed to the different features of the sidewalls of their nanopores. That is, the existence of unsaturated and saturated cations on a-Ta_2_O_5_ and a-SiO_2_, respectively. On the other hand, the presence of H_2_O is found to enhance the diffusion of Cu ions along the nanopores in both cases of a-Ta_2_O_5_ and a-SiO_2_. This can be attributed to the drastic weakening of the interaction between Cu ions and a-Ta_2_O_5_/a-SiO_2_ nanopores after H_2_O adsorption.

## 2. Theoretical method

All calculations are performed using the Vienna *ab initio* simulation package (VASP) [14,15]. A plane wave basis set with a cutoff energy of 400 eV is used. A projector augmented-wave (PAW) [16] method and a generalized gradient approximation (PW91) [17] are adopted to describe the electron-ion and electron-electron interactions, respectively. To construct the surface structures of amorphous Ta_2_O_5_, molecular dynamics (MD) simulations are carried out at room temperature using the NVT ensemble (constant number, volume, and temperature). The time steps are set at 3 fs and 1 fs for the systems without and with H_2_O, respectively. The structure is sampled up to 9 ps to obtain a sufficiently equilibrated structure. Considering the large cell size, only the Г point is used during the MD simulation for the Brillouin-zone integration. The structure optimization is performed with 2 × 2 × 1 *k*-points. The convergence criteria adopted here assumes that the maximum force acting on each atom is smaller than 0.05 eV/Å.

To investigate the diffusion behaviors of Cu ion on a-Ta_2_O_5_ surface, again MD simulation can be employed as have done in a recent study of the moisture effect on the diffusion of ions in a novel CO_2_ sorbent [18]. In the present study, however, the climbing-image nudged elastic band (CI-NEB) method [19] is employed using 2 × 2 × 1 *k*-points because realistic atomic structures of nanopores are difficult to obtain as mentioned in the next section. To examine the interaction strength between Cu or H_2_O and a-Ta_2_O_5_/a-SiO_2_ surface, the adsorption energy is defined as following: *E*_ads_ = *E*_MOx-Cu/H2O_ – *E*_Cu/H2O_ – *E*_MOx_, where *E*_MOx-Cu/H2O_, *E*_Cu/H2O_, and *E*_MOx_ denote the energies of a-Ta_2_O_5_/a-SiO_2_ surface with the adsorbed Cu or H_2_O, the pure Cu or H_2_O, and the pure a-Ta_2_O_5_/a-SiO_2_ surface, respectively.

## 3. Results and discussion

### 3.1 Atomic structure of a-Ta_2_O_5_ nanopore without water

**Figure 1.**
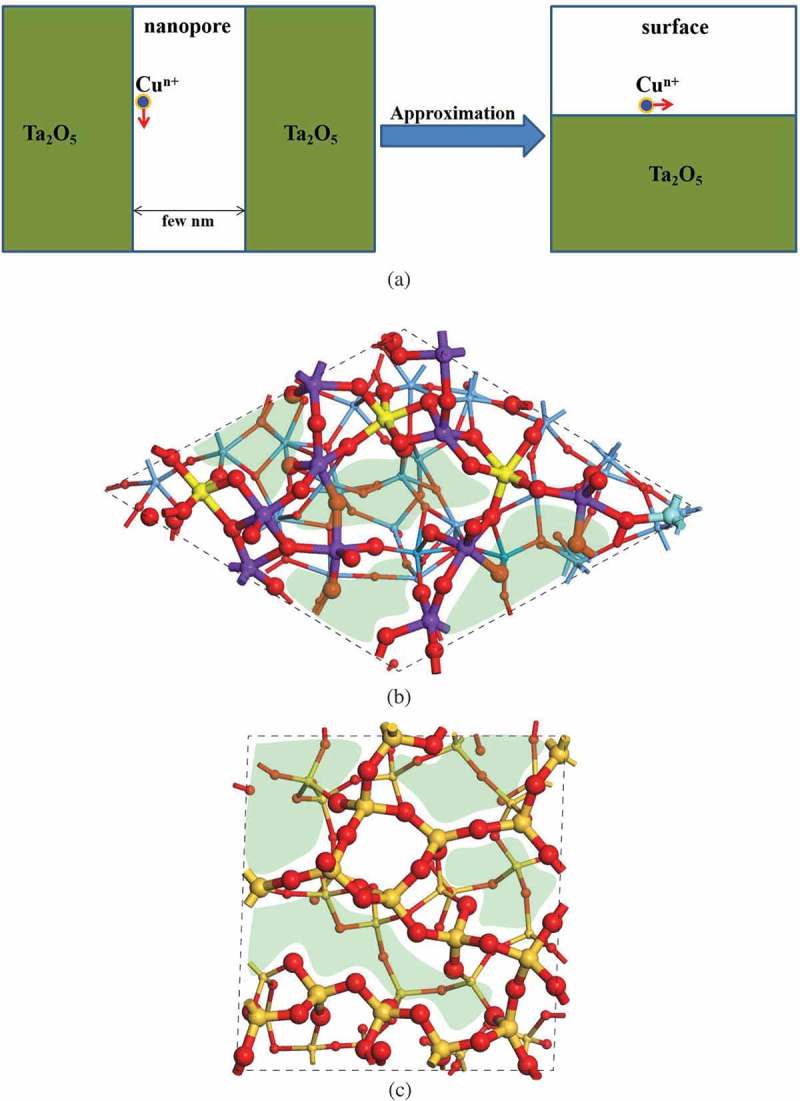
(a) Schematic of the diffusion of Cu in Ta_2_O_5_ nanopore, and the atomic structures of amorphous (b) Ta_2_O_5_ and (c) SiO_2_ surfaces, respectively. The atoms and bonds on the Ta_2_O_5_ and SiO_2_ surfaces are displayed by using ball and stick model with relatively large sizes as compared with those in the other region. The yellow, purple, and cyan balls in (b) represent the Ta atoms bonding with 6, 5, and 4 O atoms, respectively. The green regions in (b) and (c) represent the void spaces on the surfaces.

To examine the diffusion behaviors of Cu ions in a-Ta_2_O_5_-based ECM device, first a structure model of a-Ta_2_O_5_ nanopore is constructed. Experimental studies suggest that the diameters of the nanopore structures in a-Ta_2_O_5_ are about several nm [6,20,21], which are too large to be treated using first-principles simulation directly. Accordingly, we need to construct an approximate a-Ta_2_O_5_ nanopore model that can reproduce the actual Cu ion diffusion behaviors within available computational resources. Since the interaction between the two sidewalls several nm apart from each other can be negligible, the diffusion of Cu ions along the sidewall of a-Ta_2_O_5_ nanopore could be viewed as the diffusion on the surface of a-Ta_2_O_5_ as schematically shown in Figure 1(a). Therefore, we adopt a-Ta_2_O_5_ surface model to investigate the diffusion behavior of Cu ions in Cu/a-Ta_2_O_5_/Pt, instead of the a-Ta_2_O_5_ nanopore structure.

In our previous work, we constructed the a-Ta_2_O_5_ bulk model by using molecular dynamics (MD) simulation [22]. The structural features of as-generated a-Ta_2_O_5_ is consistent with the experimental data. To construct the a-Ta_2_O_5_ surface model, the a-Ta_2_O_5_ bulk model is cleaved with O-termination, which has been proven to be more stable than the Ta-terminated one [23]. Subsequently, structure optimization and MD simulation are performed at the room temperature. As shown in Figure 1(b), the as-generated a-Ta_2_O_5_ surface model (with the chemical composition of Ta_40_O_100_) possesses a relatively large surface area (14.64 × 14.86 Å^2^) with a slab thickness about 11.20 Å in average.

As discussed in our previous study [22], most of the Ta atoms in the bulk a-Ta_2_O_5_ are saturated by bonding with six O atoms. In the case of the surface region, on the other hand, most of Ta atoms are in the unsaturated forms, for example, Ta_2_O_5_ (see Figure 1(b)). This point is discussed in more detail below.

In the a-Ta_2_O_5_ bulk structure, most of the O atoms bond with two Ta atoms [22]. As a result, in the freshly cleaved a-Ta_2_O_5_ surface, most of the O atoms are coordinated with only one Ta atom. During the structure relaxation, we have observed the following trends of structural change on the a-Ta_2_O_5_ surface: (i) two adjacent O atoms with single coordination tends to bond together to form O_2_, and leave away from the system; (ii) TaO_6_ components tend to change into TaO_5_ by breaking one Ta-O bond in the subsurface region. As a result, TaO_5_ components become predominant on the a-Ta_2_O_5_ surface. In the case of a-SiO_2_, on the other hand, the surface structure has been successfully constructed in a previous theoretical study [24] as shown in Figure 1(c), where all the O atoms on the a-SiO_2_ surface are coordinated with more than one Si atoms. The Si atoms on the surface and in the bulk region are all in their saturated form (SiO_4_). In the present study, we adopt this model in our calculations.

### 3.2 The atomic structure of a-Ta_2_O_5_ surface covered with water

**Figure 2.**
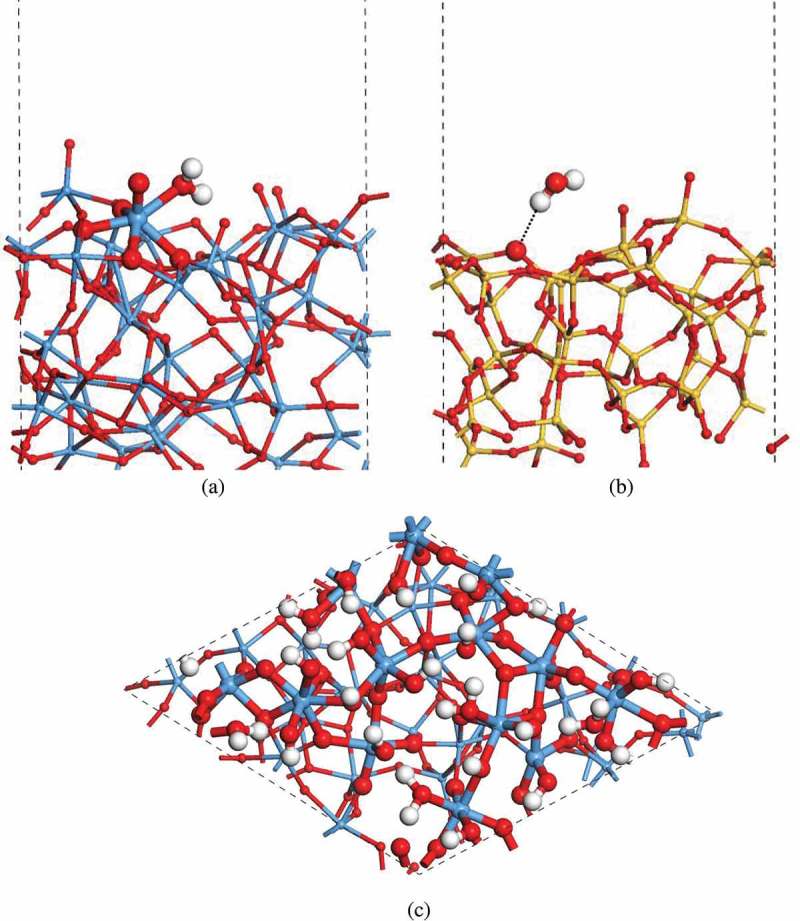
The adsorption structures of a single H_2_O molecule on amorphous (a) Ta_2_O_5_ and (b) SiO_2_ surfaces, respectively. (c) The structure of amorphous Ta_2_O_5_ surface covered by a thin layer of H_2_O.

The experimental work suggests that a small amount of H_2_O molecules tend to adhere to the sidewalls of a-Ta_2_O_5_ and a-SiO_2_ nanopores. The desorption of H_2_O takes place only at high temperature (~350°C) on a-Ta_2_O_5_ surface, but at room temperature on a-SiO_2_ surface [12,13]. To understand this point better, we have examined the adsorption of a single H_2_O molecule on the surface of a-Ta_2_O_5_ and a-SiO_2_. In doing so, all the possible adsorption sites have been considered because various chemical environments must appear on the surface of amorphous material (for details, see Figures S1 and S2 in Supporting Information). Our calculations show that a H_2_O molecule prefers to adsorb on an unsaturated Ta atom on the surface of a-Ta_2_O_5_, and it forms an O-Ta bond as seen in Figure 2(a) and Figure S1. The calculated averaged adsorption energy for such adsorption structures is about −0.94 eV, which means that the interaction between H_2_O and a-Ta_2_O_5_ surface is strong. Thus, the desorption process can only occur at high temperatures. On the other hand, in the case of a-SiO_2_, we have found that a H_2_O molecule weakly adsorbs on the a-SiO_2_ surface by forming hydrogen bonds (H∙∙∙O) as shown in Figure 2(b) and Figure S2. The calculated adsorption energy for such adsorption structures is about −0.35 eV in average. As a result, facile desorption of H_2_O from a-SiO_2_ surface is expected.

In the experiments, the sidewall of a-Ta_2_O_5_ nanopore is covered with a thin layer of H_2_O molecules [6]. To model such a situation, 10 H_2_O molecules are initially placed on a surface of a-Ta_2_O_5_ so as to form O-Ta bonds. Then the system is relaxed (with the other surface fixed) by MD simulation for 6 ps at the room temperature, and structure optimization is done subsequently. It is found that the H_2_O molecules on a-Ta_2_O_5_ surface tend to split into OH groups and H atoms during the structure relaxation. The OH group tends to adsorb on the undercoordinated Ta atom, and the H atom tends to bond with the adjacent O atom by forming another OH group. This is consistent with the fact that a large amount of OH groups was detected in the previous experimental study [6]. To achieve a full OH termination on a-Ta_2_O_5_ surface, another 8 H atoms are added to the isolated O atoms to form the OH groups. The structure relaxations (including MD simulation for 3 ps and optimization) are carried out subsequently. As shown in Figure 2(c), the as-generated a-Ta_2_O_5_-H_2_O surface model possesses a surface area of 13.89 × 14.26 Å^2^. The average slab thickness is 12.42 Å. It has a chemical composition of Ta_40_O_110_H_28_. Compared with the pure a-Ta_2_O_5_ surface, the O atoms on a-Ta_2_O_5_-H_2_O surface are stabilized by the formation of O-H×××O hydrogen bonds, and there are no O-O bonds. As a result, the Ta atoms on the a-Ta_2_O_5_-H_2_O surface are hydroxylated and possess one or two hydroxyl groups. The predominant chemical components of the Ta atoms on the surface region have changed from TaO_5_ (at pure a-Ta_2_O_5_ surface) into TaO_6_ (at a-Ta_2_O_5_-H_2_O surface). Accordingly, we can say that the adsorption of H_2_O molecules could further stabilize the surface structure of a-Ta_2_O_5_ by saturating the Ta and O atoms on the surface.

### 3.3 The diffusion of cu ions on a-Ta_2_O_5_ surface without and with water

Using the above a-Ta_2_O_5_ surface models, next we examine the diffusion behavior of Cu ion on the a-Ta_2_O_5_ surface. To do this, we first consider all the possible adsorption sites of a single Cu ion on the a-Ta_2_O_5_ surface without and with the presence of H_2_O. The possible diffusion pathways between the adjacent Cu adsorption sites are then calculated.

**Figure 3.**
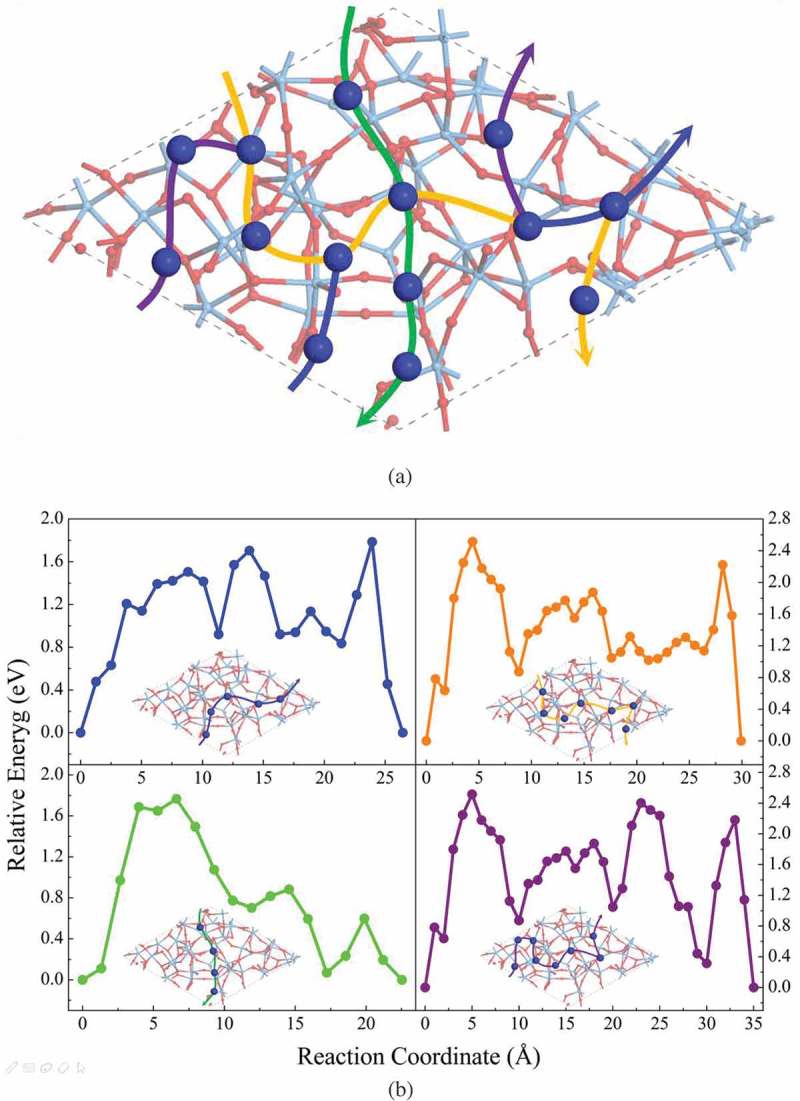
(a) The adsorption sites, the diffusion paths, and (b) the diffusion barriers of Cu on pure amorphous Ta_2_O_5_ surface.

In the case of bare a-Ta_2_O_5_ surface, total 14 Cu adsorption sites have been identified as shown in Figure 3(a). We have found that a single Cu ion prefers to locate itself between the two O atoms in the void spaces of pure a-Ta_2_O_5_ surface (for details, see the supporting information (Figure S3)). The calculated average adsorption energy of Cu ion on the bare a-Ta_2_O_5_ surface is −1.88 eV, which indicates a strong interaction between the Cu and the surface. Then, we calculate the possible diffusion pathways between two adjacent Cu adsorption sites, and four paths across the whole a-Ta_2_O_5_ surface as identified in Figure 3(b). In each path, at least one of the diffusion steps between two adjacent Cu adsorption sites has the energy barrier higher than 1.40 eV, with the maximum value of about 2.20 eV. It is to be noted that one of the authors of this work and his collaborators recently examined the diffusion barriers of Cu ion in the bulk a-Ta_2_O_5_ (with the volume of 1447.46 Å^3^) using a neural network interatomic potential fitted to density functional theory calculation data. That work reported barrier values ranging from 0.68 eV to 1.79 eV [25] for paths connecting a site in a supercell, and an equivalent one in the adjacent supercell. From calculations presented in [25] and in this work, we conclude that the diffusion of Cu ions in pure a-Ta_2_O_5_ (both the bulk region and the nanopore of a-Ta_2_O_5_) is quite difficult. In fact, experimental study in [26] has shown that the a-Ta_2_O_5_ could act as the barrier layer for the Cu ions diffusion within the temperature range of 450–600°C. It should be noted that this temperature range is higher than the desorption temperature of H_2_O from a-Ta_2_O_5_ (~350°C) [6]. Thus, the barrier property in that experiment must be ascribed to that of the pure a-Ta_2_O_5_, which further confirms our findings. Another experimental study [27] showed that the coordination number of a diffusing Cu with O atoms in a-Ta_2_O_5_ at 350°C is about 1.8 ~ 2.0. This is consistent with our theoretical result that the Cu ion prefers to bond with two O atoms on the bare surface of a-Ta_2_O_5_.

**Figure 4.**
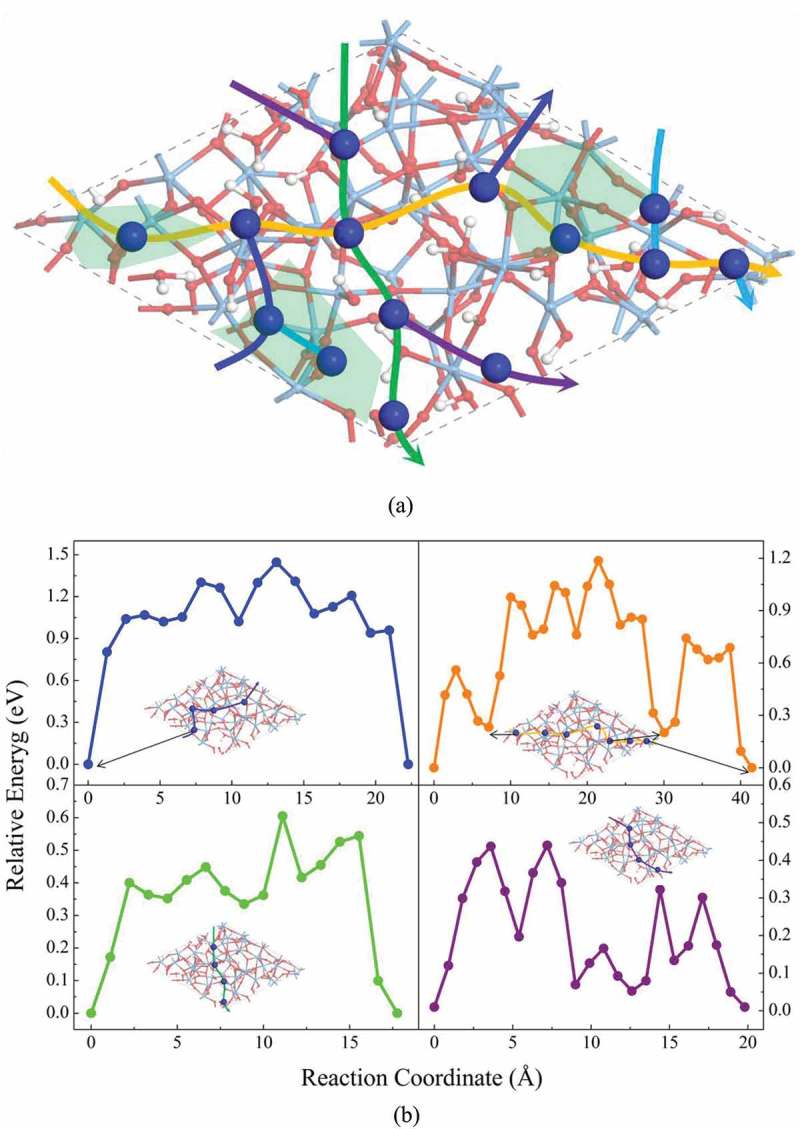
(a) The adsorption sites, the diffusion paths, and (b) the diffusion barriers of Cu on amorphous Ta_2_O_5_ surface covered by H_2_O. The green regions in (a) respect the Ta_2_O_5_ surface with low H_2_O coverage area.

In the case of a-Ta_2_O_5_ surface covered with H_2_O molecules, 14 Cu adsorption sites have been identified as shown in Figure 4(a) and Figure S4. The calculated average adsorption energy is −0.69 eV, which is obviously lower than that of pure a-Ta_2_O_5_ surface (−1.88 eV). Four diffusion paths have been considered as shown in Figure 4(b). Here, the lowest diffusion barrier is estimated to be 0.43 eV, which agrees excellently with the value estimated in a recent experimental study (0.40 eV) [27]. In addition, the estimated diffusion barrier (0.43 eV) of Cu ions on a-Ta_2_O_5_ surface covered with H_2_O is much lower than that in the case of bare a-Ta_2_O_5_ surface (>1.40 eV), which further confirms the experimental result that H_2_O molecules in a-Ta_2_O_5_ matrix could enhance Cu ion diffusion in Cu/a-Ta_2_O_5_/Pt device [6]. Accordingly, the simulation results based on our constructed models can reproduce the experimental results well.

Now, let us discuss the atomic details of the diffusion of Cu ion on a-Ta_2_O_5_ surface covered with H_2_O. After examining all the Cu adsorption structures, we have found that the adsorption energies and structures of Cu ion on a-Ta_2_O_5_ strongly depend on the H_2_O coverage on a-Ta_2_O_5_ surface. As shown in the supporting information (Figure S4), the Cu adsorption energies (−1.19 eV in average) on a-Ta_2_O_5_ surface with low H_2_O coverage area are lower than those on the surface with high H_2_O coverage (−0.43 eV in average). In the low H_2_O coverage area, Cu ions tend to be irreversibly trapped into the H_2_O layer, forming two Cu-O bonds. In this case, to escape from the trap, Cu ions need to overcome a high energy barrier (about 1.00 eV, see Figure 4(b)). In the high H_2_O coverage area, on the other hand, Cu ions prefer to bond with only one O atom. Its location is on the top of H_2_O layer. As a result, the diffusion of Cu ion on the surface of H_2_O layer is much easier since only one Cu-O bond needs to be broken in each diffusion step.

It should be noted that, in the cases of high H_2_O contents, several more H_2_O layers will adsorb on the a-Ta_2_O_5_ surface, and the diffusion of Cu ions are still on the surface of topmost H_2_O layer. Therefore, it is expected that the diffusion behaviors of Cu ions on a-Ta_2_O_5_ surface with high H_2_O contents will be similar to the case with a single H_2_O layer. Actually, Tsuruoka and Valov et al.’s experimental studies [6,9] have shown that the operation voltages of the Cu/Ta_2_O_5_/Pt device, which are strongly related to the diffusion behaviors of Cu ions, are not changed by increasing the ambient H_2_O pressure. In addition, the diffusion of Cu and H and/or O atoms may coexists. This possibility would be worth considering. This would need significantly more computations, as there are variety of possible cases. This is left as a future task. It is also left as a future task to build a mathematical model to describe the vapor pressure dependence of diffusion behaviors, such as the one constructed for an anion exchange absorbent for CO_2_ capture [28].

### 3.4 The diffusion of Cu ions on a-SiO_2_ surface without and with water

Finally, we would like to discuss the diffusion behaviors of Cu ions on a-SiO_2_ surface. As seen in Figure 1(c), there are several void spaces on the bare a-SiO_2_ surface due to the low atomic density of this material. Our results show that Cu ion prefers to locate itself in such a void space of a-SiO_2_ surface while forming two Cu-O bonds. The calculated adsorption energies for the cases of such spaces are lower than −3.16 eV. It is to be noted that, in spite of different characters of Ta and Si atoms on bare a-SiO_2_ and a-Ta_2_O_5_ surfaces, the chemical environments of O and the adsorbed Cu ion are similar on the two amorphous surfaces in the sense that the Cu has two Cu-O bonds. Thus, an irreversible trapping of Cu ion on the bare a-SiO_2_ surface is expected. With the presence of H_2_O, the void space of a-SiO_2_ surface will be filled with the H_2_O, and Cu ion will diffuse along such H_2_O layer. Similar to the case of a-Ta_2_O_5_, it is expected that the diffusion of Cu ion on a-SiO_2_ surface covered with H_2_O will be much easier than that of bare a-SiO_2_ surface.

## 4. Conclusions

In this work, the atomic structures and the diffusion behaviors of Cu ion on a-Ta_2_O_5_ and a-SiO_2_ nanopores with and without H_2_O are discussed by using the first-principles simulations. Our results reveal that the Ta and Si atoms on the sidewalls of a-Ta_2_O_5_ and a-SiO_2_ nanopores are in under- and full-coordination with O atoms, respectively. As a result, the H_2_O molecules tend to strongly adsorb in the a-Ta_2_O_5_ nanopore with forming O-Ta bonds, while the interaction is weak between H_2_O and a-SiO_2_ nanopore with the formation of H∙∙∙O hydrogen bonds. This result could well explain the experimental observation that the desorption of H_2_O from a-Ta_2_O_5_ could occur only at high temperatures, while such process could easily take place at lower temperatures in the case of a-SiO_2_. Without the presence of H_2_O, the Cu ions tend to get irreversibly trapped in the void spaces on the sidewalls of a-Ta_2_O_5_ and a-SiO_2_ nanopores by forming two Cu-O bonds. With the presence of H_2_O, the adsorption strength of Cu ions in a-Ta_2_O_5_ and a-SiO_2_ nanopores dramatically weaken, since the H_2_O fills in the void spaces in a-Ta_2_O_5_ and a-SiO_2_ nanopores. As a consequence, the diffusion of Cu ions could be enhanced by the presence of H_2_O in a-Ta_2_O_5_ and a-SiO_2_ nanopores. Our results would be helpful to understand the switching mechanisms of Cu/Ta_2_O_5_/Pt and Cu/SiO_2_/Pt resistance switches.
