# Supplementary material for: Moisture effect on the diffusion of Cu ions in Cu/Ta2O5/Pt and Cu/SiO2/Pt resistance switches: a first-principles study
Source: Sci Technol Adv Mater. 2019 Jun 3;20(1):580–8. doi: 10.1080/14686996.2019.1616222 (PMC6567064; doi:10.1080/14686996.2019.1616222)
Supplement: Supplemental Material [file TSTA_A_1616222_SM1030.pdf]

Supporting Information

Moisture effect on the diffusion of Cu ions in Cu/Ta_2O_5/Pt and Cu/SiO_2/Pt resistance switches: a first-principles study

Bo Xiao,*[†,‡] Satoshi Watanabe[†]

[†]Department of Materials Engineering, University of Tokyo, Tokyo 113-8656, Japan

[‡]Laboratory of Theoretical and Computational Chemistry, School of Chemistry and Chemical Engineering, Yantai University, Yantai 264005, China

Figure S1: The stable structures and corresponding adsorption energies for the adsorption of a single H_2O on pure a-Ta_2O_5 surface. To obtain these structures, all the possible adsorption sites, including on the top of Ta or O atom on the a-Ta_2O_5 surface, have been considered.

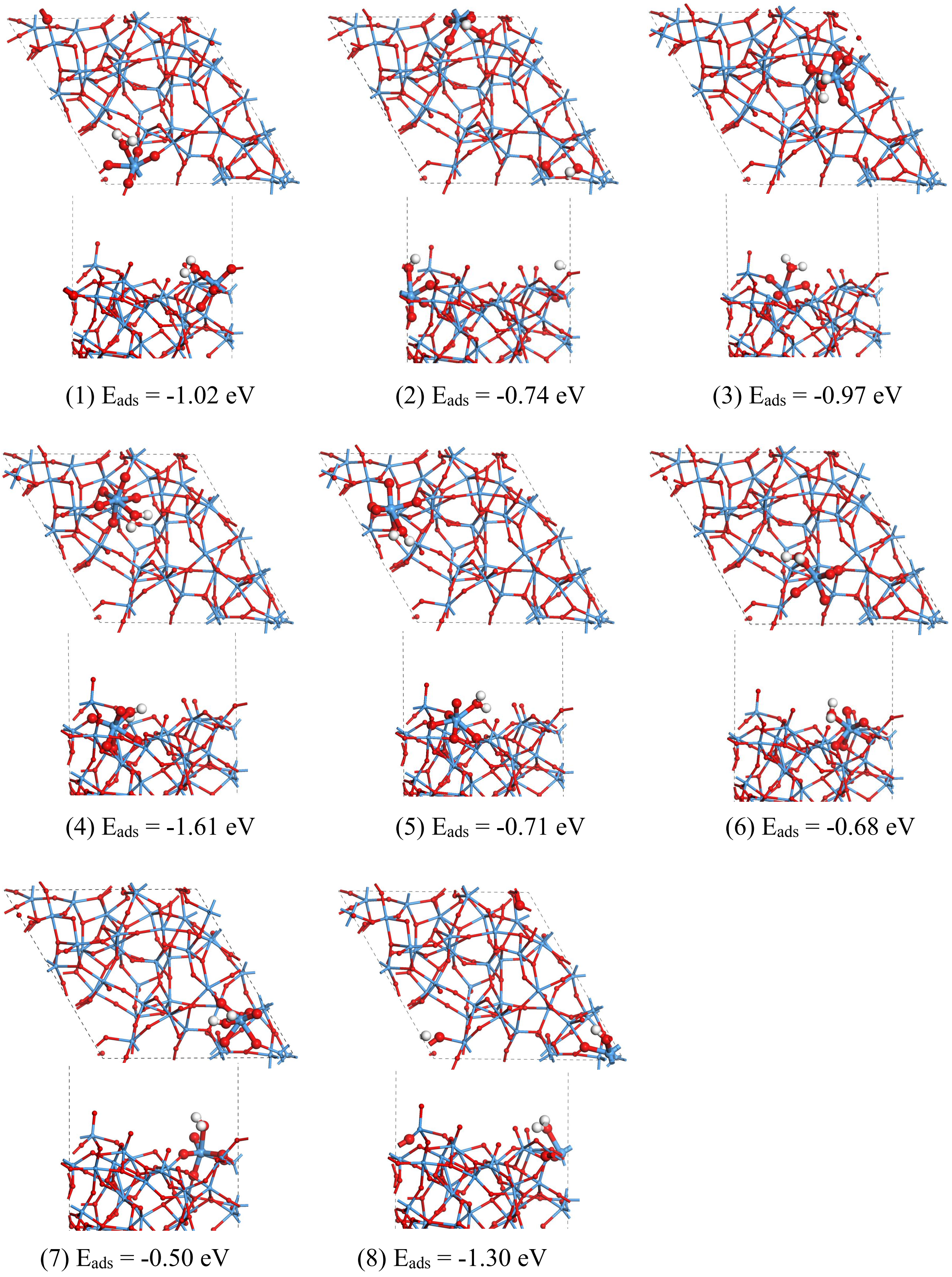

Figure S2: The stable structures and corresponding adsorption energies for the adsorption of a single H_2O on pure a-SiO_2 surface. To obtain these structures, all the possible adsorption sites, including on the top of Si or O atom on the a-SiO_2 surface, have been considered.

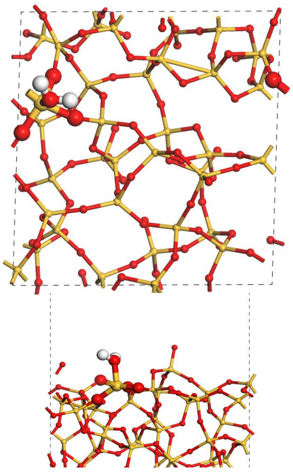

(1) E_{ads} = -0.91 eV

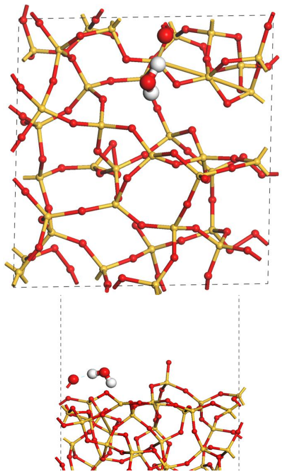

(2) E_{ads} = -0.43 eV

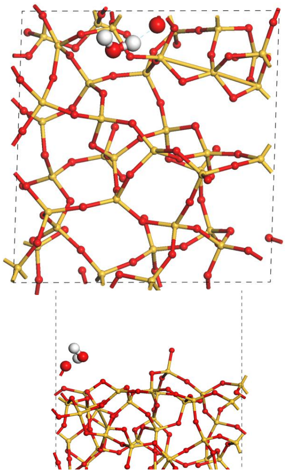

(3) E_{ads} = -0.38 eV

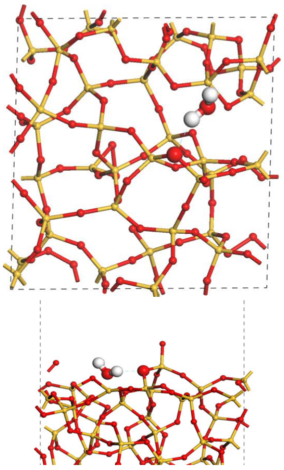

(4) E_{ads} = -0.36 eV

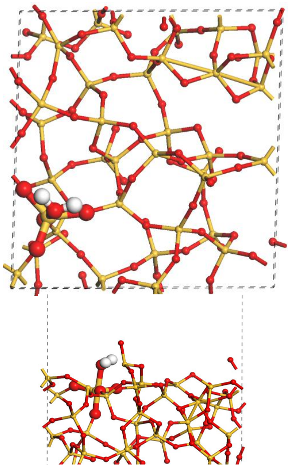

(5) E_{ads} = -0.33 eV

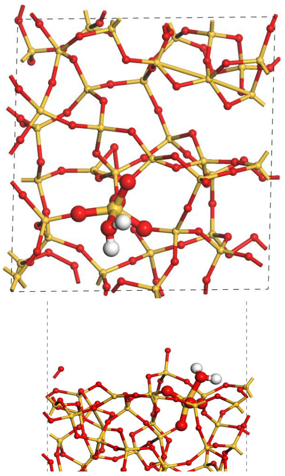

(6) E_{ads} = -0.31 eV

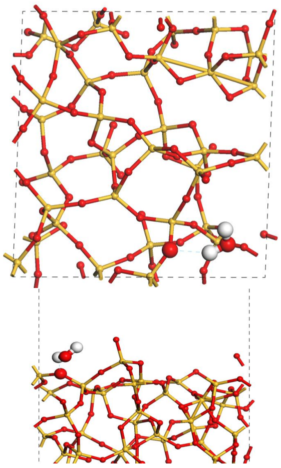

(7) E_{ads} = -0.18 eV

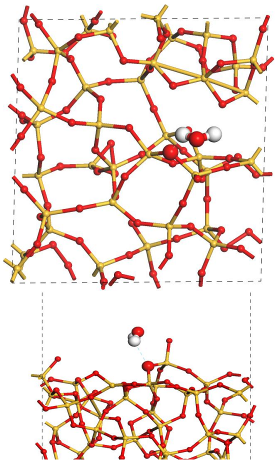

(8) E_{ads} = -0.18 eV

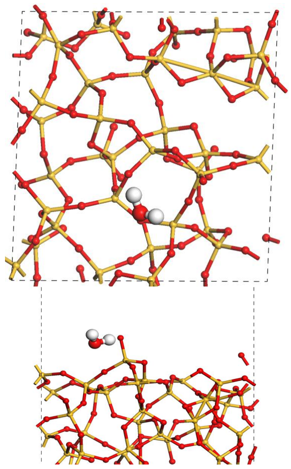

(9) E_{ads} = -0.11 eV

Figure S3: The structures and corresponding adsorption energies for the adsorption of Cu on pure a-Ta_2O_5 surface

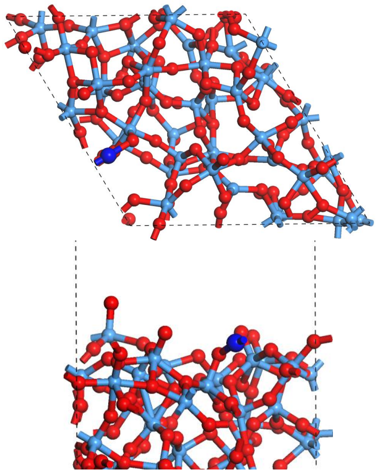

(1) E_{ads} = -2.89 eV

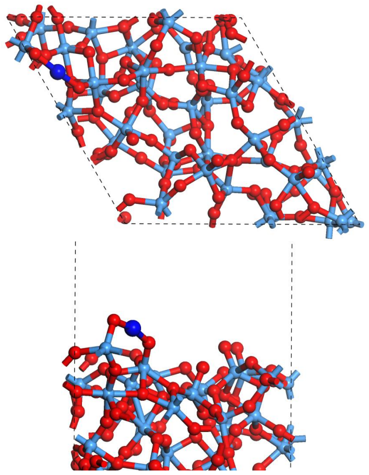

(2) E_{ads} = -2.88 eV

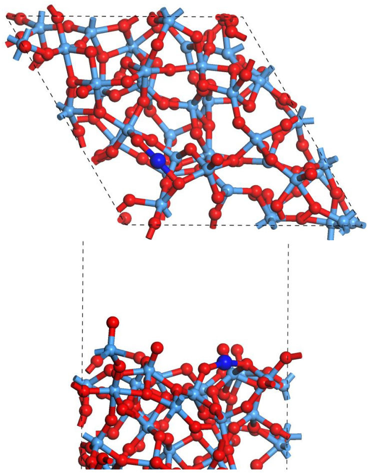

(3) E_{ads} = -2.24 eV

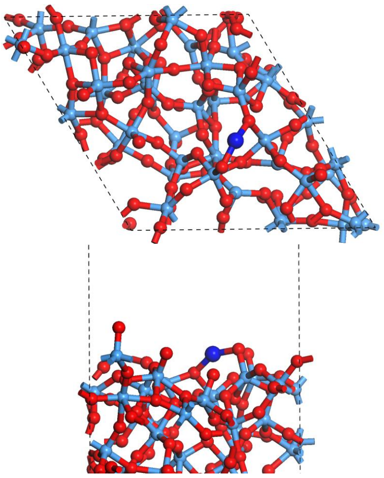

(4) E_{ads} = -2.14 eV

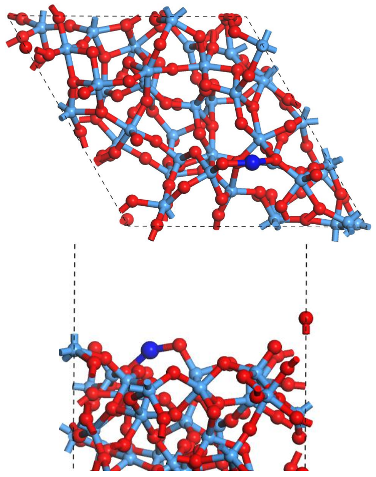

(5) E_{ads} = -2.15 eV

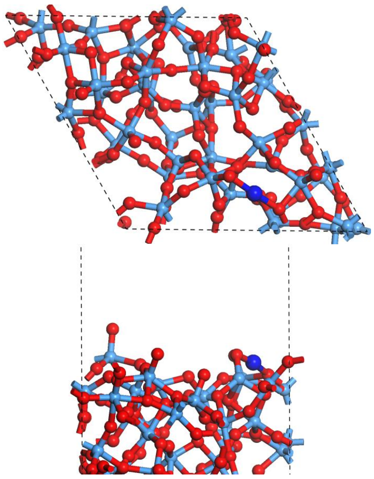

(6) E_{ads} = -2.06 eV

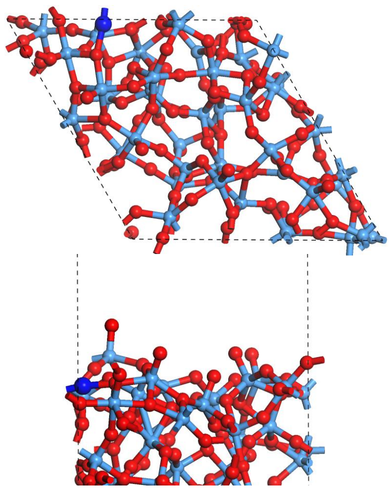

(7) E_{ads} = -2.05 eV

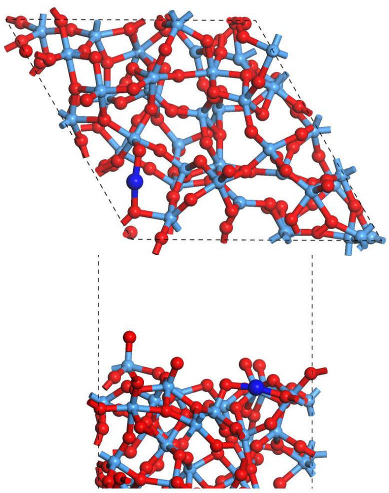

(8) E_{ads} = -2.30 eV

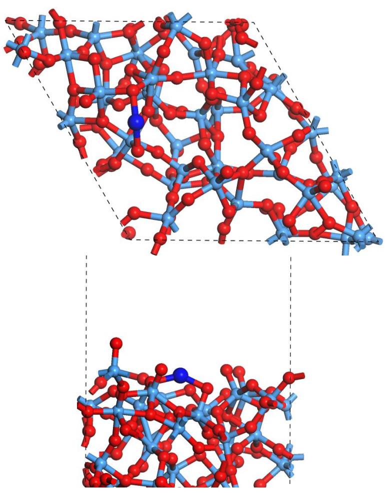

(9) E_{ads} = -1.85 eV

Figure S3 (continued): The structures and corresponding adsorption energies for the adsorption of Cu on pure a-Ta_2O_5 surface

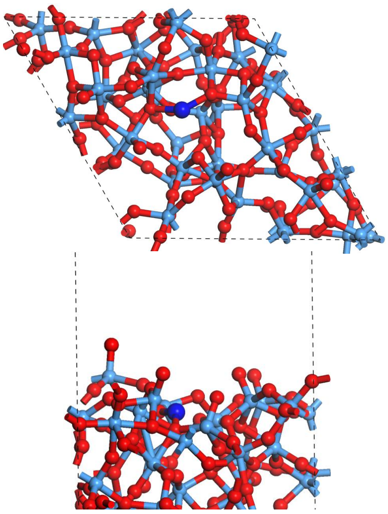

(10) E_{ads} = -1.70 eV

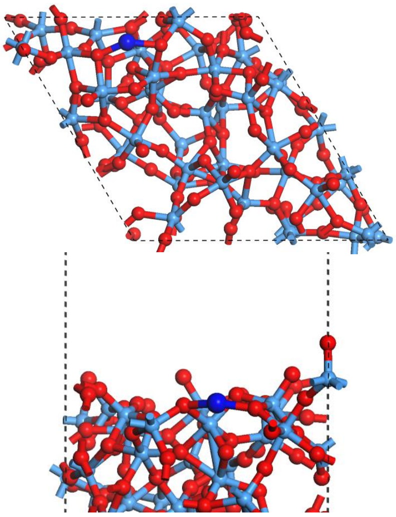

(11) E_{ads} = -1.39 eV

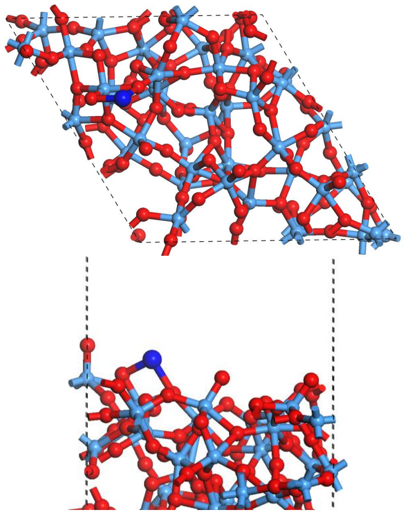

(12) E_{ads} = -1.16 eV

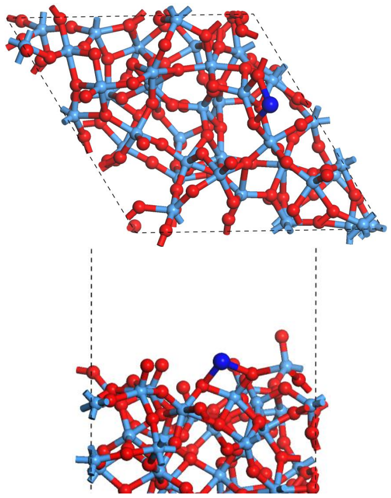

(13) E_{ads} = -0.88 eV

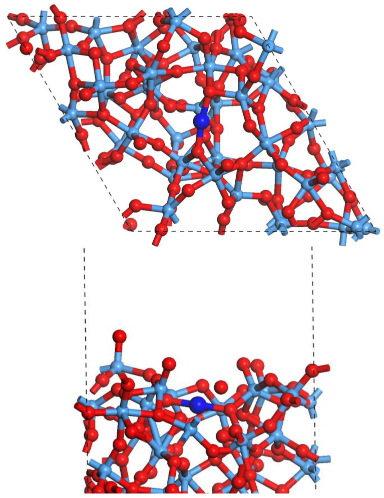

(14) E_{ads} = -0.65 eV

Figure S4: The structures and corresponding adsorption energies for the adsorption of Cu on a-Ta_2O_5-H_2O surface

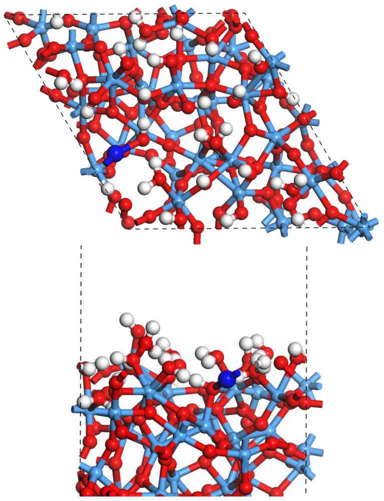

(1) E_{ads} = -1.45 eV

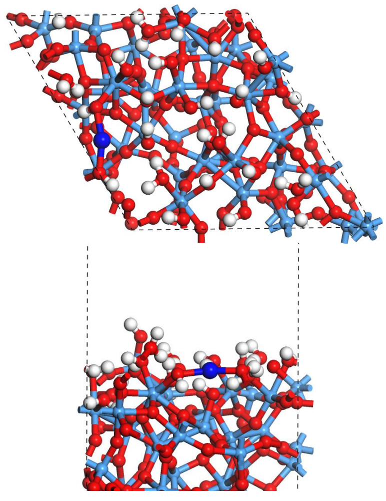

(2) E_{ads} = -1.43 eV

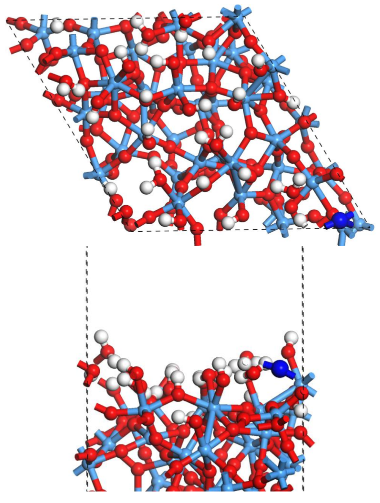

(3) E_{ads} = -1.19 eV

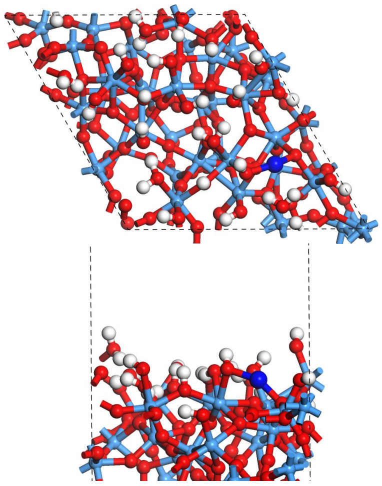

(4) E_{ads} = -0.96 eV

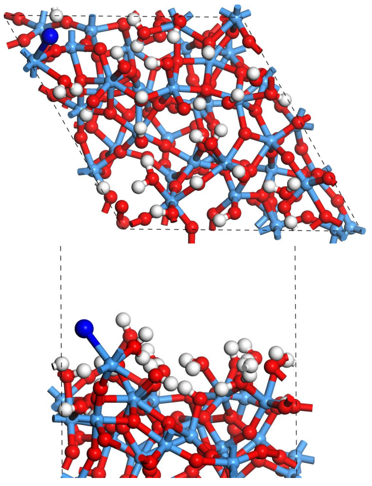

(5) E_{ads} = -0.92 eV

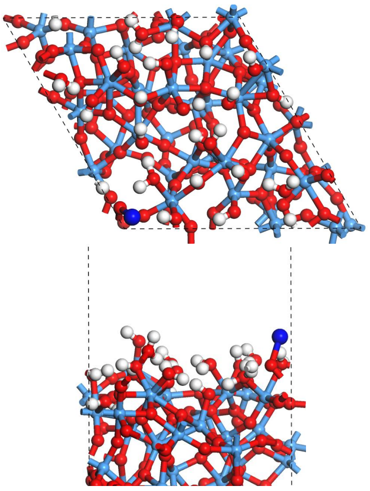

(6) E_{ads} = -0.72 eV

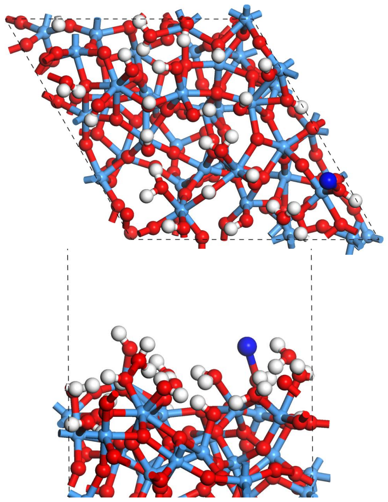

(7) E_{ads} = -0.55 eV

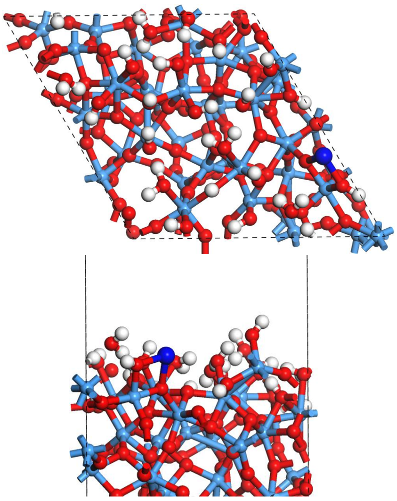

(8) E_{ads} = -0.52 eV

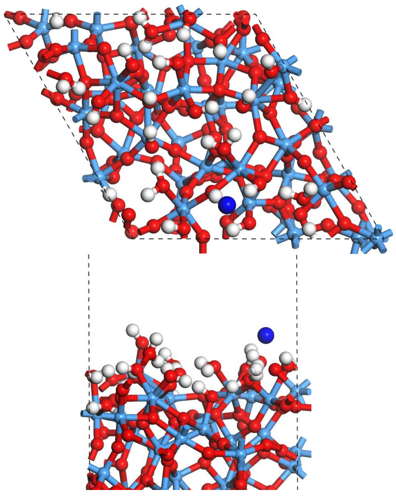

(9) E_{ads} = -0.46 eV

Figure S4 (continued): The structures and corresponding adsorption energies for the adsorption of Cu on a-Ta_2O_5-H_2O surface

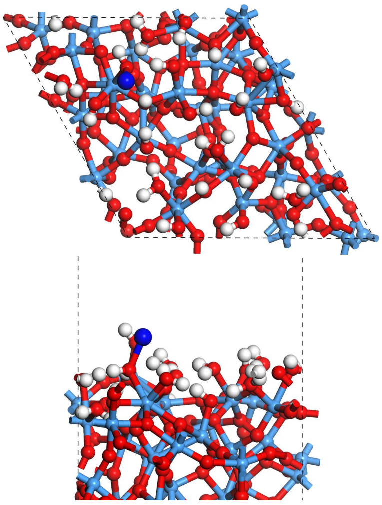

(10) E_{ads} = -0.41 eV

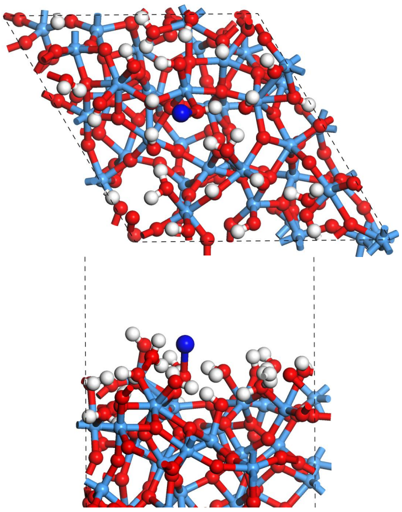

(11) E_{ads} = -0.39 eV

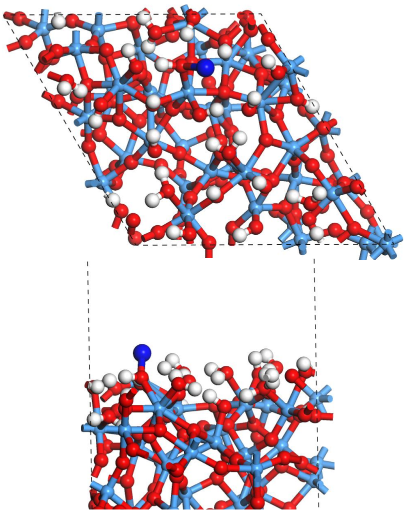

(12) E_{ads} = -0.37 eV

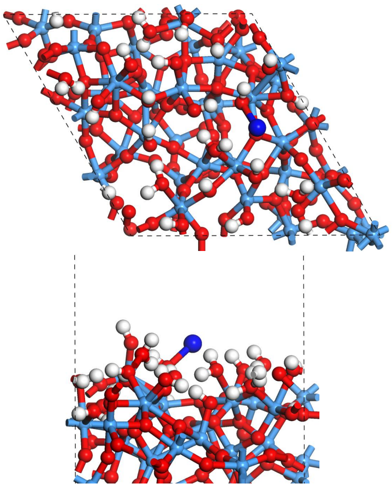

(13) E_{ads} = -0.34 eV

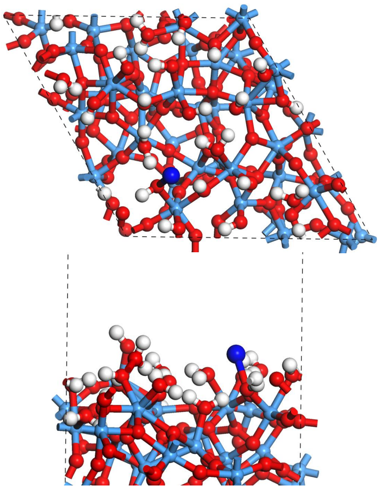

(14) E_{ads} = -0.27 eV
